# *TGFA* and *IRF6* Contribute to the Risk of Nonsyndromic Cleft Lip with or without Cleft Palate in Northeast China

**DOI:** 10.1371/journal.pone.0070754

**Published:** 2013-08-06

**Authors:** Yongping Lu, Qiang Liu, Wei Xu, Zengjian Li, Miao Jiang, Xuefu Li, Ning Zhao, Wei Liu, Yu Sui, Chao Ma, Wenhua Feng, Weitian Han, Jianxin Li

**Affiliations:** 1 Key Laboratory of Reproductive Health, Liaoning Province Research Institute of Family Planning, Shenyang, China; 2 Department of Oral-Maxillofacial Surgery and Plastic Surgery, School of Stomatology, China Medical University, Shenyang, China; Boston Children's Hospital, United States of America

## Abstract

Nonsyndromic cleft lip with or without cleft palate (NSCL/P) are common birth defects with a complex etiology. Multiple interacting loci and possible environmental factors influence the risk of NSCL/P. 12 single nucleotide polymorphisms (SNPs) in 7 candidate genes were tested using an allele-specific primer extension for case-control and case-parent analyses in northeast China in 236 unrelated patients, 185 mothers and 154 fathers, including 128 complete trios, and 400 control individuals. *TGFA* and *IRF6* genes showed a significant association with NSCL/P. In *IRF6*, statistical evidence of an association between rs2235371 (p = 0.003), rs2013162 (p<0.0001) and NSCL/P was observed in case-control analyses. Family based association tests (FBATs) showed over-transmission of the C allele at the rs2235371 polymorphism (p = 0.007). In *TGFA*, associations between rs3771494, rs3771523 (G3822A), rs11466285 (T3851C) and NSCL/P were observed in case-control and FBAT analyses. Associations between other genes (*BCL3*, *TGFB3*, *MTHFR*, *PVRL1* and *SUMO1*) and NSCL/P were not detected.

## Introduction

Nonsyndromic cleft lip with or without cleft palate (NSCL/P) is one of the most common birth defects, affecting 1 in 500 to 1000 births worldwide [1]. In China, this common malformation occurs at a rate of 1.6 per 1000 live births. NSCL/P presents a significant public health problem because treatment requires comprehensive surgical, orthodontic, phoniatric and psychological management [2].

Progress has been made toward defining genetic variations involved in oral-facial cleft by utilizing gene discovery techniques, including genome wide linkage, association mapping and candidate gene approaches. Positive associations between 14 interacting genes and NSCL/P have been reported [3]. Carinci et al. reviewed genes and available loci in the literature and suggested that 6p24, 2p13, 19q13.2, 4q, *Msx1*, *IRF6*, *PVRL1*, *TP73L*, 13q33, 1q34 and *SUMO1* are related to oral clefts [4]. Single nucleotide polymorphism (SNP) genotyping using competitive allele-specific PCR were conducted in 12 candidate genes, including *CLPTM1*, *CRISPLD2*, *FGFR2*, *GABRB3*, *GLI2*, *PTCH1*, *RARA*, *RYK*, *SATB2*, *SUMO1*, *TGFA*, and *IRF6*, and reported that *PTCH1*, *SUMO1*, and *TGFA* contribute to nonsyndromic oral clefts [5]. Through multiple stages involving positional cloning, candidate gene sequencing and developmental gene expression analysis, FOXE1 had been identified as a major disease gene for NSCL/P [6].

Most recently, several genome-wide association studies (GWAS) reported a major susceptibility locus for NSCL/P on 8q24.21 and other genes [7]. A case-control genome-wide association study (GWAS) in Germany found significant evidence of association with markers in 8q24.21 [8], and a US case-control GWAS confirmed this region, with rs987525 being the most significant marker in both studies [9]. Mangold and colleagues subsequently used an expanded dataset from Europe and identified additional loci at chromosomes 10q25 (VAX1, ventral anterior homeobox 1) and 17q22 (NOG, noggin) that achieved genome-wide significance [10]. Beaty and colleagues performed a GWAS using a case-parent trio from Europe, the United States, China, Taiwan, Singapore, Korea and the Philippines and found that SNPs in two genes not previously associated with CL/P (ABCA4 on chromosome 1p22.1 and MAFB on 20q12) achieved genome-wide significance, and three potential candidate genes (PAX7 on 1p36, VAX1 on 10q25.3 and NTN1 on 17p13) had one or more SNPs near genome-wide significance [11]. These novel loci have subsequently been replicated in independent studies [12]–[14]. Leslie et al. resequenced the ARHGAP29 and identified a nonsense variant, a frame shift variant, and fourteen missense variants were overrepresented [15]. The GWAS Meta analysis study based on the two GWAS identifies six new risk loci [16].

Transforming growth factor alpha (*TGFA*) belongs to a large family of proteins that regulate cell proliferation, differentiation, migration and apoptosis. Ardinger et al. first reported an association between the Taq I variant in *TGFA* and NSCL/P [17]. *TGFA* has since been extensively investigated for linkage, association and gene-environment interactions with inconsistent results. The mutations (C3827T, G3822A, and T3851C) in 3′untranslated conserved regions were reported to be associated with oral-facial clefts [18]. Sull et al. recently tested 17 SNPs in a region surrounding *TGFA* on chromosome 2p13 and reported over-transmission of rs3771494 as the minor allele without considering the parent-of-origin in four populations [19].

The interferon regulatory factor 6 (*IRF6*) gene, located on chromosome 1q32.3-q41, has been frequently studied and is strongly associated with oral-facial cleft risk. This gene encodes interferon regulatory factor 6, which is a key element in oral and maxillofacial development. Scapoli et al. selected four SNPs based on Zucchero et al. [20] and reported that rs2013162 and rs2235375 have strong linkage disequilibrium with NSCL/P in an Italian family [21]. Srichomthong et al. suggested that *IRF6* rs2235371 (V274I) is responsible for 16.7% of the genetic contribution to CL/P [22]. Large studies in different populations [23]–[26] have provided evidence that *IRF6* is important in the etiology of NSCL/P.

Many studies have suggested that other genes and loci are associated with NSCL/P[27]–[34]. Due to NSCL/P genetic heterogeneity in different populations, we investigated the contribution of previously reported candidate genes *TGFA*, *IRF6*, *BCL3*, *TGFB3*, *MTHFR*, *PVRL1*, and *SUMO1* for NSCL/P in northeast China (Table 1).

**Table 1:** Candidate SNP function and MAF in northeast China.

gene	chromosome	SNPs	SNP function	Allele	MAF
*TGFA*	2p13	rs3771494	3'UTR	T/c	0.238
*TGFA*	2p13	rs3771523	3'UTR	G/a	0.275
*TGFA*	2p13	rs11466285	3'UTR	T/c	0.143
*TGFA*	2p13	rs1058213	3'UTR	C/t	0.334
*IRF6*	1q32.2	rs2235371	in 7 extron	C/t	0.363
*IRF6*	1q32.2	rs2013162	in 5 extron	C/a	0.446
*BCL3*	19p13-q12	rs1046881	in 9 extron	T/a	0.242
*TGFB3*	14q21-q24	rs3917201	in 5 intron	G/a	0.494
*MTHFR*	1p36	rs13306554	in 4 extron	C/t	0.195
*MTHFR*	1p36	rs1801131	in 7 extron	A/c	0.225
*PVRL1*	11q23.3	rs104894281	in 3 extron	G/a	0.273
*SUMO1*	2q32-q36	rs7580433	in 2 intron	G/a	0.158

MAF, minor allele frequency.

Erdogan et al. used a novel allele-specific primer elongation microarray to test SNPs in human mitochondrial DNA (mtDNA) [35]. Jagomagi et al. performed SNP genotyping using an arrayed primer extension technique [36]. In this research, we designed a similar microarray to test the risk of 12 SNPs in 7 genes that may contribute to NSCL/P in a population from northeast China. This microarray is a high throughout, efficient, accurate and convenient method for test SNPs, and the current study of northeast China populations was supplement for the previous association study between candidate genes and NSCL/P.

## Materials and Methods

### Ethics

This study was approved by the ethics committee at the Liaoning Province Research Institute of Family Planning. Written informed consent was obtained from all study participants. Parents or legal guardians provided written consent on behalf of minors.

### Patients and Families

We evaluated 236 unrelated patients with NSCL/P, their parents (185 mothers and 154 fathers, including 128 complete trios), and 400 control individuals. All participants were recruited from Stomatology Hospital of China Medical University in northeast China between 2008 and 2012. All patients underwent a pre-operation examination and questionnaire, and photographs were taken to record the shape of the lips, alveolar ridge, and hard and soft palates to diagnose cleft lip and palate. The patients had a physical examination to document the shape of the skull, eyes, nose, chin, neck, chest, feet, and hands to exclude syndromes that may be associated with cleft lip and palate, such as Albert syndrome, Edward syndrome, and Pierre Robin syndrome. All the controls were recruited from healthy volunteers. Controls with a family history of clefts and other anomalies were excluded based on a questionnaire that evaluated the medical history of the patients' family for three generations. A physical examination was also performed on the controls, particularly regarding movement of the lips and soft palate to detect occult deformities. A five milliliter peripheral blood sample was collected. DNA was obtained from blood samples with a TIANamp Blood DNA kit (TianGen) and used as the template for PCR amplification.

### Allele Special Primer Extension Microarray Preparation

A total of 24 oligonucleotide tags used as probe captures were obtained from www.genome.wi.mit.edu. The 3′-amino modified tags (Table 2) were synthesized by Nanjing Genescript Biological Technology Company. Tags were suspended at a concentration of 30 µM in 0.3 M carbonate buffer (pH 8.0) and spotted onto slides in duplicate using a Nano-Plotter 2.1 arrayer (GeSiM Germany) at a rate of ten hits per spot in a humidified chamber (60% relative humidity). After spotting, the microarrays were incubated at room temperature overnight in a humid chamber and broiled at 60°C for 1 hour. Microarrays were rinsed in dH2O and dried.

**Table 2:** SNP probes and allele-specific extension primer sequences.

Gene	SNPs	allele	Tag	Allele-specific extension primers
*TGFA*	rs3771494	T	TTCAGTGTATGACGACCAGAGCGTT-NH2	AACGCTCTGGTCGTCATACACTGAATTTGTTAAGAATGGCAGATTT
		C	AACGTCCACGCAGGCTCTCATAGTG-NH2	CACTATGAGAGCCTGCGTGGACGTTTTTGTTAAGAATGGCAGATTC
*TGFA*	rs3771523	G	CACAAGGAGGTCAGACCAGATTGAA-NH2	TTCAATCTGGTCTGACCTCCTTGTGAGCTGTATCCTCTAACCACG
		A	ACACATACGATTCTGCGAACTTCAA-NH2	TTGAAGTTCGCAGAATCGTATGTGTAGCTGTATCCTCTAACCACA
*TGFA*	rs11466285	T	GATGCCAATCCACGTGGTGTAATTCC-NH2	GGAATTACACCACGTGGATTGGCATCACCAGCCCAACATCTTCCAT
		C	CTGCAATACGGTGAGCGGTATATCC-NH2	GGATATACCGCTCACCGTATTGCAGACCAGCCCAACATCTTCCAC
*TGFA*	rs1058213	C	TTACAGGATGTGCTCAACAGACGTT-NH2	AACGTCTGTTGAGCACATCCTGTAATATCCTCTAACCACGAGACC
		T	GGACAGACAGTGGCTACGGCTCAGTT-NH2	AACTGAGCCGTAGCCACTGTCTGTCCTATCCTCTAACCACGAGACT
*IRF6*	rs2235371	C	CGTTCAGATAGAGCCACTGATGAGG-NH2	CCTCATCAGTGGCTCTATCTGAACGTCCCGTCAGCCTGGAGCAGC
		T	ATGAGGTACACCAAGCGATTCATCC-NH2	GGATGAATCGCTTGGTGTACCTCATTCCCGTCAGCCTGGAGCAGT
*IRF6*	rs2013162	C	GCTTATCGGAAGTGAACGAATACTT-NH2	AAGTATTCGTTCACTTCCGATAAGCAAGATGAGCTGGATCAGTCC
		A	GATAGGATTAGAAGGTCGAACCGTT-NH2	AACGGTTCGACCTTCTAATCCTATCAAGATGAGCTGGATCAGTCA
*BCL3*	rs1046881	T	TGTGCCGTTCCACTTCTGATATTCC-NH2	GGAATATCAGAAGTGGAACGGCACAGAGGGGCCCCCCTGCCCTGT
		A	GCAAGCGCGTTAGTCATGGTGGTAG-NH2	CTACCACCATGACTAACGCGCTTGCGAGGGGCCCCCCTGCCCTGA
*TGFB3*	rs3917201	G	TAACATCTGCAATCGCGGCCAGTAC-NH2	GTACTGGCCGCGATTGCAGATGTTATCAACAGAGGGTCCCTGATG
		A	TACTCCACATCCATGCTGTAACGCC-NH2	GGCGTTACAGCATGGATGTGGAGTATCAACAGAGGGTCCCTGATA
*MTHFR*	rs13306554	C	TGCTCTCGGAATATCAATGAAGGAA-NH2	TTCCTTCATTGATATTCCGAGAGCAAGCCCATAAGCTCCCTCCAC
		T	GGCTATCTATCGGCTTATTAGTACTTG-NH2	CAAGTACTAATAAGCCGATAGATAGCCAGCCCATAAGCTCCCTCCAT
*MTHFR*	rs1801131	A	GGTATAGATATAGAGTCGGCATACA-NH2	TGTATGCCGACTCTATATCTATACCGAGGAGCTGACCAGTGAAGA
		C	CCATGTCATACACCGCCTTCAGAGC-NH2	GCTCTGAAGGCGGTGTATGACATGGGAGGAGCTGACCAGTGAAGC
*PVRL1*	rs104894281	G	GTATCTGCATATGATGTCTGACGCTGGC-NH2	GCCAGCGTCAGACATCATATGCAGATACCTCCCAGTGTGGTATCCTGG
		A	ACACTCTGGCTGATGGACGCAATCT-NH2	AGATTGCGTCCATCAGCCAGAGTGTCTCCCAGTGTGGTATCCTGA
*SUMO-1*	rs7580433	G	AACAGAGAGGTTCGAAGTGAGCGAA-NH2	TTCGCTCACTTCGAACCTCTCTGTT CACTCCAGCCTGCCAACAAGAG
		A	GCTATACAGGCCAACATTGAGTTAT-NH2	ATAACTCAATGTTGGCCTGTATAGCCACTCCAGCCTGCCAACAAGAA

### Allele-specific Primer Design

Each allele-specific primer was composed of a reverse complement of the tag (com-tag) at forward and backward sequences. For each SNP, two allele-specific primers were designed to match the two SNP alleles (Table 2). In addition, a com-tag labeled with Cy3 was synthesized as a positive control.

### Multiplex PCR and Allele-specific Primer Extension

PCR was amplified using the Multiplex PCR Assay Kit (Takara) in a 20 µL volume (Multiplex PCR mix2 10 µL, Multiplex PCR mix1 0.2 µL, 0.2 µM primers, and 50 ng DNA). Thermocycling was performed with an initial 60 second denaturation at 94°C followed by 35 cycles of 30 seconds at 94°C, 90 seconds at 57°C, 90 seconds at 72°C, and final extension at 72°C for 10 minutes. The PCR product was used as a template for multiplex allele-specific primer extension. To remove excess dNTPs and primers, 2 units of shrimp alkaline phosphatase (Takara) and 4 units of EXO I (Takara) were added to 10 µL of the PCR product, followed by incubation at 37°C for 30 min and 96°C for 10 min. A total of 10 µL of treated PCR products were added to 5 µL ASPE containing 1×Thermopol reaction buffer, 80 µM each of dATP, dGTP, and dTTP (Takara), 40 µM Cy3-dCTP (GE), 0.2 units of Vent exo-DNA polymerase (New England Biolabs), and 25 nM allele-specific primers. Extension conditions were as follows: initial 3 minutes at 94°C, 35 cycles of 94°C for 30 seconds, 60°C for 30 seconds, 72°C for 1 minute, and a final extension at 72°C for 5 minutes.

### Hybridization, Scanning of Microarrays and Data Analyses

To prepare Cy3-labeled single strand products, 10 µL allele-specific extension products were added to a 10 µL mixture containing 1.33×SSC, 0.067% SSC, 0.033 mg/ml salmon sperm DNA, and 50 nM Cy3-labeled com-tag. The mix was denatured at 94°C for 10 minutes and cooled for 5 minutes on ice. Hybridization was performed using a hybridization solution in a hybridization chamber at 60°C for 2 hours. Hybridization was followed by two washes in 2×SSC/0.1% SDS (preheated to 50°C) for 5 minutes, washes in 0.2×SSC/0.1% SDS for 1 minute, then rinsed in dH_2_O and dried by centrifugation for 3 minutes at 500 rpm.

Microarrays were scanned at 100-µm resolution using a GenePix 4000B scanner, and TIFF images were imported into GenePix 6.0 software (Axon Instruments, USA). For each allele, the mean pixel intensity was subtracted from the mean background intensity. Alleles with a mean intensity lower than the cutoff (mean background +1000) were excluded. SNP genotypes were identified by the allelic fraction (AF), which was calculated as follows: AF = allele B/allele A+allele B. AF values >0.6, <0.4, and between 0.4 and 0.6 represented allele B homozygotes, allele A homozygotes, and heterozygotes, respectively. Microarray results can be acquired from Gene Expression Omnibus (GSE45770 accession number).

### Statistical Methods

Hardy-Weinberg equilibrium (HWE) was used to assess all SNPs. Case-control statistical analysis was performed using the SPSS 17.0 statistical software package (http://www-03.ibm.com/software/products/us/en/spss-stats-standard/). Odds ratios and 95% confidence intervals were calculated for the case and control groups. The Family Based Association Test (FBAT) package (http://www.biostat.harvard.edu/fbat/default.html) was used to test for over-transmission of the target alleles in case-parent trios. Linkage disequilibrium (LD) was estimated using haploview (http://www.broad.mit.edu/mpg/haploview) based on the genotypes of the control samples. *TGFA* and *IRF6* haplotypes were calculated using the FBAT software package.

## Results

### Genotype and Case-control Comparisons

**Figure 1:**
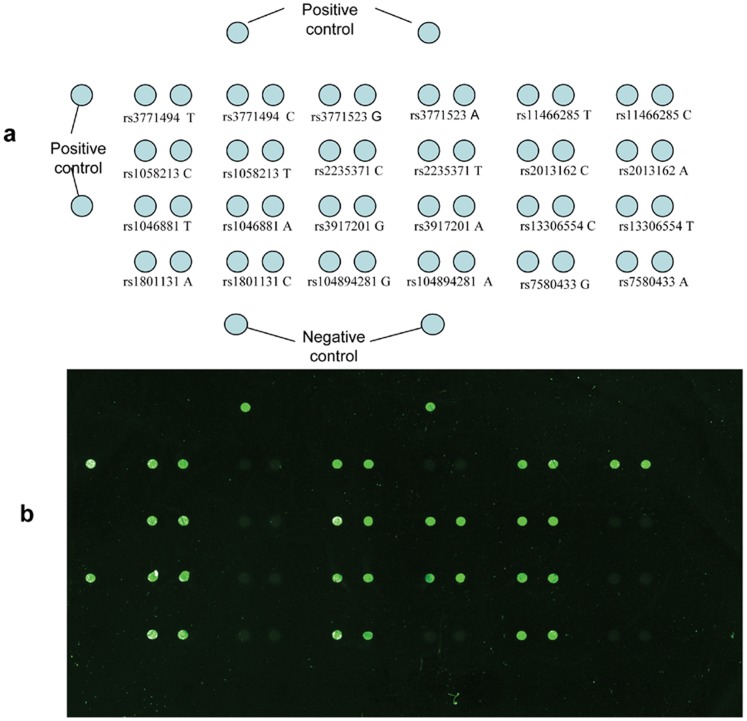
Spot array and result of the genechip. a: Spot arrays printed on the slide. b: Results of allele-specific primer extension microarray after hybridization.

Figure 1 shows the SNP microarray results. Each SNP AF was calculated as described, and the SNP genotypes were clearly identified.

To ensure accuracy, 50 samples were run in duplicate, and 10% of all samples were sequenced directly. SNP genotypes were highly reproducible (99.5%), and direct sequenced demonstrated agreement with the microarray (99.6%).

Hardy-Weinberg equilibrium was assessed for the 12 SNPs. There was no evidence of deviation from HWE for any of the SNPs. Genotype distribution between cases was compared with controls (Table 3).

**Table 3:** Genotype distribution for 12 SNPs and associations between SNPs and NSCL/P.

GENE	rs no.	genotype	cases(frequency)	controls(frequency)	OR(95%CI)	X^2^	P^a^
*TGFA*	rs3771494	TT	123(0.52)	269(0.67)	referent	–	–
		TC	82(0.35)	112(0.28)	1.601(1.122–2.286)	6.767	**0.009**
		CC	31(0.13)	19(0.05)	3.568(1.94–6.564)	18.31	**0.000**
		TC+CC	113(0.48)	131(0.17)	1.886(1.356–2.624)	14.37	**0.000**
*TGFA*	rs3771523	GG	156(0.66)	313(0.78)	referent	–	–
		GA	71(0.3)	80(0.2)	1.673(1.155–2.425)	7.479	**0.006**
		AA	9(0.04)	7(0.02)	2.424(0.887–6.626)	3.159	0.076
		GA+AA	80(0.34)	87(0.22)	1.734(1.213–2.478)	9.218	**0.002**
*TGFA*	rs11466285	TT	151(0.64)	305(0.76)	referent	–	–
		TC	71(0.3)	84(0.21)	1.707(1.178–2.475)	8.056	**0.005**
		CC	14(0.06)	11(0.03)	2.571(1.14–5.79)	5.508	**0.019**
		TC+CC	85(0.36)	95(0.24)	1.807(1.271–2.569)	11	**0.001**
*TGFA*	rs1058213	CC	146(0.62)	260(0.65)	referent	–	–
		CT	75(0.32)	120(0.3)	1.113(0.782–1.584)	0.354	0.552
		TT	15(0.06)	20(0.05)	1.336(0.664–2.688)	0.661	0.416
		CT+TT	90(0.38)	140(0.35)	1.145(0.82–1.598)	0.632	0.427
*IRF6*	rs2235371	CC	122(0.52)	152(0.38)	referent	–	–
		CT	96(0.41)	180(0.45)	0.664(0.471–0.937)	5.455	**0.02**
		TT	18(0.7)	68(0.17)	0.33(0.18–0.584)	15.33	**0.000**
		CT+TT	123(0.52)	248(0.62)	0.618(0.448–0.852)	8.652	**0.003**
*IRF6*	rs2013162	CC	57(0.24)	156(0.39)	referent	–	–
		CA	132(0.56)	200(0.5)	1.806(1.242–2.627)	9.678	**0.002**
		AA	47(0.2)	44(0.11)	2.923(1.754–4.873)	17.54	**0.000**
		CA+AA	179(0.76)	244(0.61)	2.008(1.402–2.876)	14.69	**0.000**
*BCL3*	rs1046881	TT	115(0.49)	184(0.46)	referent	–	–
		TA	105(0.445)	180(0.45)	0.933(0.668–1.305)	0.163	0.686
		AA	16(0.065)	36(0.09)	0.711(0.377–1.34)	1.12	0.29
		TA+AA	121(0.51)	216(0.54)	0.896(0.649–1.237)	0.444	0.505
*TGFB3*	rs3917201	GG	81(0.345)	132(0.33)	referent	–	–
		GA	104(0.44)	184(0.46)	0.921(0.638–1.329)	0.193	0.66
		AA	51(0.215)	84(0.21)	0.989(0.634–1.543)	0.002	0.963
		GA+AA	155(0.655)	268(0.67)	0.943(0.671–1.324)	0.116	0.733
*MTHFR*	rs13306554	CC	115(0.49)	212(0.53)	referent	–	–
		CT	99(0.42)	152(0.38)	1.201(0.855–1.687)	1.113	0.292
		TT	22(0.09)	36(0.06)	1.127(0.633–2.006)	0.164	0.685
		CT+TT	121(0.51)	188(0.44)	1.186(0.86–1.637)	1.084	0.298
*MTHFR*	rs1801131	AA	143(0.605)	260(0.65)	referent	–	–
		AC	79(0.335)	120(0.3)	1.197(0.844–1.698)	1.017	0.313
		CC	14(0.06)	20(0.05)	1.273(0.624–2.596)	0.441	0.506
		AC+CC	93(0.395)	140(0.35)	1.208(0.866–1.684)	1.242	0.265
*PVRL1*	rs104894281	GG	172(0.73)	290(0.725)	referent	–	–
		GA	52(0.22)	96(0.24)	0.913(0.62–1.344)	0.212	0.646
		AA	12(0.05)	14(0.035)	1.445(0.653–3.196)	0.835	0.361
		GA+AA	64(0.27)	110(0.275)	0.981(0.684–1.408)	0.011	0.917
*SUMO1*	rs7580433	GG	148(0.63)	260(0.65)	referent	–	–
		GA	66(0.28)	118(0.295)	0.983(0.684–1.412)	0.009	0.924
		AA	22(0.09)	22(0.055)	1.757(0.941–3.28)	3.189	0.074
		GA+AA	88(0.37)	140(0.35)	1.104(0.79–1.543)	0.338	0.561

a Chi-square analysis.

Bold values represent significance at p<0.05.

There was no evidence of genotypic association with the risk of NSCL/P in northeast China for the following SNPs: *TGFA* rs1058213 (C3827T), *BCL3* rs1046881, *TGFB3* rs3917201, *MTHFR* rs13306554 (C667T) and rs1801131 (A1298C), *PVRL1* rs104894281 (G546A), and *SUMO1* rs7580433. However, a statistically significant increased risk of NSCL/P was observed with *TGFA* rs3771494, rs3771523 (G3822A), and rs11466285 (T3851C) and *IRF6* rs2235371 (V274I) and rs2013162.

For *TGFA* rs3771494, ORs for cases with TC+CC, TC and CC genotypes compared with TT homozygotes were 1.601 (95% CI, 1.122–2.286, p = 0.009), 3.568 (95% CI, 1.94–6.564, p<0.0001), and 1.886 (95% CI, 1.356–2.624, p = 0.001), respectively. For rs3771523, the OR of the GA heterozygote compared with the GG homozygote was 1.673 (95% CI, 1.155–2.425, p = 0.006). Although the OR of the AA genotype compared with GG was not statistically significant (p = 0.076), the OR of GA+AA compared with the GG homozygote was significant (p = 0.002). For rs11466285, a significant association was observed between patients and controls (p<0.05).

For *IRF6* rs2235371 and rs2013162, a risk of NSCL/P was observed in all of the genetic models evaluated (Table 3). The ORs of CT, TT, and CT+TT compared with the CC genotype were 0.664 (95% CI, 0.471–0.937, p = 0.02), 0.33 (95% CI, 0.18–0.584, p = 0.001), and 0.618 (95% CI, 0.448–0.852, p = 0.003), respectively. For *IRF6* rs2013162, the ORs of CA, AA, and CA+AA compared with the CC genotype were 1.806 (95% CI, 1.242–2.627, p = 0.002), 2.923 (95% CI, 1.754–4.873, p<0.0001), and 2.008 (95% CI, 1.402–2.876, p<0.0001), respectively.

### FBAT

FBAT showed strong associations between *TGFA* rs3771494, rs3771523 (G3822A), and rs11466285 (T3851C) and *IRF6* rs2235371 (V274I) and rs2013162 and NSCL/P (Table 4). These SNPs showed an over-transmission of the minor frequency alleles, with the exception of *IRF6* rs2235371, which showed under-transmission of the T allele and over-transmission of the common C allele.

**Table 4:** FBAT results for 12 SNPs in case-parent trios.

gene	SNPs	allele	Frequency	Fam#	Z	P
*TGFA*	rs3771494	T	0.735	78	−2.156	0.016
		C	0.265	78	2.156	0.016
*TGFA*	rs3771523	G	0.785	83	−2.555	0.01
		A	0.215	83	2.555	0.01
*TGFA*	rs11466285	T	0.79	65	−2.133	0.03
		C	0.21	65	2.133	0.03
*TGFA*	rs1058213	C	0.606	59	−0.237	0.812
		T	0.394	59	0.237	0.812
*IRF6*	rs2235371	C	0.68	72	2.673	0.007
		T	0.32	72	−2.673	0.007
*IRF6*	rs2013162	C	0.64	76	−3.555	0.001
		A	0.38	76	3.555	0.001
*BCL3*	rs1046881	T	0.558	62	−0.753	0.451
		A	0.442	62	0.753	0.451
*TGFB3*	rs3917201	G	0.636	67	0.297	0.767
		A	0.364	67	−0.297	0.767
*MTHFR*	rs13306554	C	0.778	76	1.115	0.265
		T	0.222	76	−1.115	0.265
*MTHFR*	rs1801131	A	0.805	81	1.482	0.138
		C	0.195	81	−1.482	0.138
*PVRL1*	rs104894281	G	0.726	64	−0.385	0.54
		A	0.274	64	0.385	0.54
*SUMO1*	rs7580433	G	0.617	57	−0.41	0.681
		A	0.383	57	0.41	0.681

Frequency: gene frequency.

Fam#: number of families.

Z: vector of the FBAT statistic.

### LD and Haplotype Analysis

Pairwise LD was measured for *TGFA* and *IRF6* (Table 5). LD analysis showed tight linkage between the SNPs in *IRF6* and *TGFA*.

**Table 5:** LD of SNPs in *TGFA* and *IRF6.*

Gene	SNP	r^2^\D'			
*TGFA*		rs3771494	rs1058213	rs11466285	rs3771523
	rs3771494	–	1	1	1
	rs1058213	0.73	–	1	1
	rs11466285	0.75	0.87	–	1
	rs3771523	0.48	0.51	0.47	–
*IRF6*		rs2235371	rs2013162		
	rs2235371	–	1		
	rs2013162	0.467	–		

D’ is above the diagonal, r^2^ is below the diagonal.

Haplotypes of the four *TGFA* SNPs (rs3771494, rs1058213, rs11466285, and rs3771523) and two *IRF6* SNPs (rs2013162 and rs2235371) were also determined. The C-G-C-C (order: rs3771494-rs3771523-rs11466285-rs1058213) and C-A (order: rs2235371-rs2013162) haplotypes showed significant associations with NSCL/P (Table 6).

**Table 6:** *IRF6* and *TGFA* haplotypes.

gene	haplotype	frequency	Fam#	z	p
*IRF6*	C-C	0.655	63	−3.25	0.36
	C-A	0.22	48	2.28	0.03
	T-A	0.07	32	1.6	0.56
*TGFA*	T-G-T-C	0.542	68	−2.24	0.35
	C-G-C-C	0.154	32	1.16	0.027
	C-G-T-C	0.121	22	0.533	0.043

IRF6, order of SNPs: rs2235371-rs2013162.

TGFA, order of SNPs: rs3771494-rs3771523-rs11466285-rs1058213.

Frequency: haplotype frequency.

Fam#: number of families.

Z: vector of the FBAT statistic.

## Discussion

We used a microarray with allele-specific primer extension to test 12 SNPs in 7 genes reported to be associated with NSCL/P and observed that SNPs in *TGFA* and *IRF6* had statistically significant associations with NSCL/P based on case-control and case-parent analyses consisting of 236 patients and 400 controls from northeast China.

The *TGFA* gene has been well studied since Ardinger et al. first reported an association between the Taq I variant and NSCL/P [17]. However, there have been conflicting results reported for different population due to study design, sample size, and *TGFA* variants used [37]. Machida et al tested SNPs in the 3′ UTR of *TGFA* and reported that there were no associations with NSCL/P [18], but Shiang et al. reported significant associations between those SNPs and cleft palate [38]. Letra et al. recently calculated the attributable fraction of high-risk alleles at *IRF6* rs2235371 and *TGFA* rs1058213 in a Brazilian population and estimated the contribution of interactions between the two genes to be approximately 1% [39]. They also evaluated *IRF6*-*TGFA* interactions in 142 case-parent trios and detected significant over-transmission of high-risk alleles to the affected child (p = 0.001). Sull et al. examined associations between *TGFA* markers and NSCL/P in case-parent trios from four populations [19]. The authors genotyped 17 SNPs and reported significant transmission of the minor allele in rs3771494 (OR = 1.59, p = 0.004).

In this study, a significant association with rs3771494 (OR = 1.88, p<0.0001) was observed in the case-control analysis. FBAT analysis also showed over-transmission of the C allele in rs3771494 (p = 0.016). These consistent results suggest that *TGFA* is strongly associated with NSCL/P in populations in northeast China. We also observed an association between rs3771523 (OR = 1.73, p = 0.002) and rs11466285 (OR = 1.81, p = 0.001) and over-transmission of the A allele in rs3771523 (p = 0.01) and the C allele in rs11466285 (p = 0.03) based on FBAT analysis. However, a significant association was not observed between rs1058213 and NSCL/P in the present study (OR = 1.14, p = 0.427), which is in accordance with previous studies. Using HBAT analysis, we observed an over-transmission of the C-G-C-C haplotype (order: rs3771494-rs3771523-rs11466285-rs1058213).

*IRF6* is the most frequently studied gene related to NSCL/P. Research has shown common alleles in *IRF6* that are associated with NSCL/P, which has been independently replicated in genome-wide association (GWA) [11], [40] and candidate gene studies [41], [42]. Animal models have also supported the role of *IRF6* in NSCL/P [43], [44]. Birnbaum et al. reported that the T allele in rs2235371 (V274I) coding for isoleucine is under-representation in NSCL/P patients compared with controls and may have a protective effect with respect to NSCL/P [40]. However, significant associations were not found between rs2013162 and NSCL/P, which differs from previous findings [21], [23], [45], [46]. Letra et al. demonstrated a significant association between the V274I polymorphism (rs2235371) with complete left cleft lip/palate (p = 0.001) in a Brazilian Caucasian population [39]. Huang et al. found a significant association between rs2235371 and rs2235375 and NSCL/P in west China, but not rs2013162 [47]. In the present study, both rs2235371 (p = 0.003) and rs2013162 (p<0.0001) showed significant differences between patients and controls. rs2013162 results are in contrast to the Huang et al. study, which may be due to population heterogeneity between west and northeast China. FBAT analysis of rs2235371 showed under-representation T allele and over-transmission of the C allele from parent to child, indicating that the minor T allele may be a protective factor in NSCL/P, which is consistent with a previous study [40]. In rs2013162, we observed an over-transmission of the A allele (minor allele) and under-transmission of the C allele (common allele). In haplotype analyses of NSCL/P trios, the C-A (order: rs2235371-rs2013162) haplotype showed significant over-transmission (p = 0.03). These results suggest that the *IRF6* gene is strongly associated with an increased risk of NSCL/P in populations in northeast China.

Small ubiquitin-related modifier (*SUMO1*) is strongly expressed in the upper lip, primary palate and MEE of the secondary palate [33]. Several studies [5], [48] have shown associations between gene variations in *SUMO1* and NSCL/P. Jia et al. reported that the C allele in rs7580433 is overtransmitted from parents to affected individuals in west China [49]. However, we did not observe an association between rs7580433 in *SUMO1* and cases and controls (p = 0.561). FBAT analysis of trios did not detect an association between rs7580433 and NSCL/P (p = 0.68).

We failed to replicate an association between candidate gene SNPs, including *BCL3*, *TGFB3*, *MTHFR* and *PVRL1*, and NSCL/P in our patient sample. This could be the result of (1) locus and allelic heterogeneity in NSCL/P, (2) selection of too few SNPs that did not span the entire gene, (3) a small number of analyzed patients and controls, and (4) lack of analysis of gene-gene and gene-environmental interactions, which can play an important role in NSCL/P etiology.

In this study, we tested 12 SNPs in 7 candidate genes using a microarray technique. This study examined associations between SNPs and NSCL/P in northeast China. We confirmed that polymorphic variants of *TGFA* and *IRF6* are strongly associated with NSCL/P in a population in northeast China. This is a supplement research for the previous studies. To understand the fully genetic architecture of these genes, additional SNP-based and resequencing studies with a large sample of patients were still needed in further study.
