# Correction: *TGFA* and *IRF6* Contribute to the Risk of Nonsyndromic Cleft Lip with or without Cleft Palate in Northeast China

**DOI:** 10.1371/journal.pone.0116257

**Published:** 2014-12-16

**Authors:** The PLOS ONE Staff

The grant number for the second funder, the Natural Science Foundation of Liaoning Province, is incorrect. The correct grant number is: 201102247. Please see the full, correct funding statement below.

The funders of Scientific and Technological Project of Liaoning Province (Grant No.2009225007-7) played an important role in study design, data analysis, preparation of the manuscript and decision to publish, the Natural Science Foundation of Liaoning Province (Grant No. 201102247) played an important role in the sample collection and diagnosis of the NSCL/P.
